# Cochlin Deficiency Protects Against Noise-Induced Hearing Loss

**DOI:** 10.3389/fnmol.2021.670013

**Published:** 2021-05-24

**Authors:** Richard Seist, Lukas D. Landegger, Nahid G. Robertson, Sasa Vasilijic, Cynthia C. Morton, Konstantina M. Stankovic

**Affiliations:** ^1^Eaton-Peabody Laboratories and Department of Otolaryngology – Head and Neck Surgery, Massachusetts Eye and Ear, Boston, MA, United States; ^2^Department of Otolaryngology – Head and Neck Surgery, Harvard Medical School, Boston, MA, United States; ^3^Department of Otorhinolaryngology – Head and Neck Surgery, Paracelsus Medical University, Salzburg, Austria; ^4^Department of Otorhinolaryngology – Head and Neck Surgery, Medical University of Vienna, Vienna, Austria; ^5^Department of Obstetrics and Gynecology and of Pathology, Brigham and Women’s Hospital, Harvard Medical School, Boston, MA, United States; ^6^Broad Institute of MIT and Harvard, Cambridge, MA, United States; ^7^Manchester Centre for Audiology and Deafness, School of Health Sciences, University of Manchester, Manchester, United Kingdom; ^8^Program in Speech and Hearing Bioscience and Technology, Harvard Medical School, Boston, MA, United States; ^9^Harvard Program in Therapeutic Science, Harvard Medical School, Boston, MA, United States

**Keywords:** cochlin, noise-induced hearing loss, conductive hearing loss, cytokines, LCCL, sterile inflammation

## Abstract

Cochlin is the most abundant protein in the inner ear. To study its function in response to noise trauma, we exposed adolescent wild-type (*Coch^+/+^*) and cochlin knock-out (*Coch*^–/–^) mice to noise (8–16 kHz, 103 dB SPL, 2 h) that causes a permanent threshold shift and hair cell loss. Two weeks after noise exposure, *Coch^–/–^* mice had substantially less elevation in noise-induced auditory thresholds and hair cell loss than *Coch*^+^*^/^*^+^ mice, consistent with cochlin deficiency providing protection from noise trauma. Comparison of pre-noise exposure thresholds of auditory brain stem responses (ABRs) and distortion product otoacoustic emissions (DPOAEs) in *Coch^–/–^* mice and *Coch*^+^*^/^*^+^ littermates revealed a small and significant elevation in thresholds of *Coch^–/–^* mice, overall consistent with a small conductive hearing loss in *Coch^–/–^* mice. We show quantitatively that the pro-inflammatory component of cochlin, LCCL, is upregulated after noise exposure in perilymph of wild-type mice compared to unexposed mice, as is the enzyme catalyzing LCCL release, aggrecanase1, encoded by *Adamts4*. We further show that upregulation of pro-inflammatory cytokines in perilymph and cochlear soft-tissue after noise exposure is lower in cochlin knock-out than wild-type mice. Taken together, our data demonstrate for the first time that cochlin deficiency results in conductive hearing loss that protects against physiologic and molecular effects of noise trauma.

## Introduction

Coagulation factor C Homolog (*COCH*) is the most highly expressed gene in the cochlea (Robertson et al., 1994, 1997; Shen et al., 2015), and its encoded protein, cochlin, is the most abundant protein detected in the inner ear (Ikezono et al., 2001; Robertson et al., 2006). Cochlin consists of an N-terminal signal peptide (SP) for secretion, an LCCL (*Limulus* factor C, Cochlin, and late gestation Lung protein) domain, followed by two vWFA (von Willebrand-factor A-like) domains, separated by intervening domains, ivd1 and ivd2. Mutations in *COCH* cause the adult-onset progressive sensorineural hearing loss (HL) and vestibular disorder, DFNA9 (Khetarpal et al., 1991). To date, there are 29 known missense and 2 in-frame deletions causative of autosomal dominant hearing loss, DFNA9. These mutations result in deleterious function of cochlin with a distinct aggregative histopathology in inner ear structures, pathognomonic for DFNA9 (Khetarpal et al., 1991; Robertson et al., 1998, 2006; Khetarpal, 2000), as well as dimerization and high-molecular-weight multimerization of mutant cochlins *in vitro* (Yao et al., 2010; Bae et al., 2014), indicative of the dominant negative properties of mutant cochlin.

To study cochlin function, we previously utilized *Coch* “knock-out” (*Coch^–/–^*) mice that lack cochlin and *Coch* “knock-in” (*Coch^*G*88*E/G*88*E*^*) mice that incorporate the human p.G88E missense mutation (Robertson et al., 2008). Both heterozygous and homozygous *Coch^*G*88*E/G*88*E*^* mice recapitulate DFNA9 hearing loss and vestibular phenotype (Robertson et al., 2008; Jones et al., 2011). However, the heterozygous knock-out mice (*Coch^+/–^*) have hearing function comparable to wild-type littermates (Jones et al., 2011). This observation in a murine model is consistent with evidence that *COCH* haploinsufficiency is not the cause of hearing loss in humans (Masuda et al., 2016). However, mice that lack cochlin (homozygous *Coch^–/–^* mice) show a trend for mild HL limited to the highest frequency tested, and no apparent histological defects (Robertson et al., 2008; Jones et al., 2011). Relevantly, mice lacking cochlin have been utilized to demonstrate a fundamental role for cochlin in protecting the cochlea from bacteria and in systemic innate immunity (Py et al., 2013; Nyström et al., 2018; Choe et al., 2019).

Based on the mild hearing loss phenotype in *Coch^–/–^* mice, we hypothesized that a more pronounced phenotype would become apparent after noise exposure. We therefore utilized the cochlin-knockout mouse model compared to wild-type (*Coch*^+/+^) and heterozygous (*Coch*^+/–^) littermates to investigate cochlin’s function and biology in noise-induced hearing loss.

## Materials and Methods

### Animals and Experimental Design

The *Coch^–/–^* mouse model backcrossed into the CBA/CaJ mouse strain (Jones et al., 2011) was bred with wild-type CBA/CaJ mice purchased from Jackson Laboratory (Bar Harbor, ME, United States). *Coch*^+/–^ mice were bred to generate *Coch*^+^*^/^*^+^, *Coch*^+/–^, and *Coch^–/–^* littermates. Genotypes were confirmed by Transnetyx company (Cordova, TN, United States) after completion of experimental procedures by researchers blinded to the genotype. Four litters of either sex were used. Cochlear function was tested by measuring ABRs and DPOAEs 2–4 days before noise exposure at 6 weeks of age (*N* = *10 Coch*^+^*^/^*^+^, *N* = *13 Coch*^+/–^, *N* = *12 Coch^–/–^* mice). Animals were then exposed to noise causing permanent threshold shift (PTS), as described below. Fourteen days after noise exposure, cochlear function was reassessed with ABRs and DPOAEs. Cochlear tissue and perilymph were collected from *Coch*^+^*^/^*^+^ mice bred homozygous (*N* = *32* mice) and *Coch^–/–^* mice (*N* = *28*).

All experimental procedures were approved by the Institutional Animal Care and Use Committee of Massachusetts Eye and Ear and conducted in accordance with the NIH Guide for the Care and Use of Laboratory Animals.

### Noise Exposure

Mice were exposed to PTS-causing octave-band noise (8–16 kHz) for 2 h at 103 dB sound pressure level (SPL) in a reverberant, acoustically transparent wire box on a rotating platform, as routine in our laboratory (Jensen et al., 2015; Landegger et al., 2019). Animals were awake and unrestrained during noise exposure. The digitally created noise using a fifth-order Butterworth filter was amplified through a power amplifier (D75A, Crown, Elkhart, IN, United States) and delivered by a loudspeaker (2446H, JBL, Los Angeles, CA, United States) coupled to an exponential horn in the roof of the box. Exposure levels were measured in each cage with a 0.25-inch Brüel & Kjaer (Naerum, Denmark) condenser microphone.

### Cochlear Function Testing

ABRs and DPOAEs were recorded as detailed previously (Seist et al., 2020). Briefly, mice were anesthetized with intraperitoneally administered ketamine (100 mg/kg) and xylazine (10 mg/kg). A small incision was made in the external ear canal to provide a clear view and access to the tympanic membrane. Two miniature earphones serving as sound sources (CDMG15008-03A, CUI, Tualatin, OR, United States) and a microphone (FG-23329-P07, Knowles, Itasca, IL, United States) coupled to a probe tube within a custom acoustic system were used to measure sound pressure near the eardrum. DPOAEs were measured as ear canal pressure in response to two tones presented into the ear canal (*f*_1_ and *f*_2_, with *f*_2/_*f*_1_ = 1.2 and *f*_1_ being 10 dB above *f*_2_) at half octave steps from *f*_2_ = 5.66 to 45.25 kHz, and in 5 dB intensity increments from 10 to 80 dB SPL. DPOAE thresholds were defined as the *f*_2_ intensity required to generate a DP response 10 dB SPL over noise floor. Five ms tone pips were used to elicit ABR, which was measured between subdermal electrodes (adjacent to the ipsilateral incision, at the vertex, and near the tail), amplified 10,000 times and filtered (0.3–3.0 kHz). A total of 512 responses were recorded for each frequency and sound level and averaged using custom LabVIEW data-acquisition software run on a PXI chassis (National Instruments Corp., Austin, TX, United States). ABR waveforms stacked from lowest to highest SPL were visually inspected to define threshold as the first level at which a repeatable waveform was visually detected.

### Cochlear Whole Mounts and Quantitative Confocal Fluorescence Immunohistochemistry

After intracardial perfusion of deeply anesthetized animals with 4% paraformaldehyde (PFA, #P6148, Sigma-Aldrich, St. Louis, MO, United States), both cochleae were extracted. The round and oval window membranes were punctured and gentle intracochlear perfusion with PFA was performed. Cochleae were post-fixed for 2 h in 4% PFA and decalcified in 0.12M EDTA (#17892, Thermo Fisher Scientific, Waltham, MA, United States) for 48 h. The spiraling cochleae were microdissected into six pieces to prepare whole mounts of the organ of Corti. These pieces were blocked with 5% normal horse serum (NHS, #16050130, Thermo Fisher Scientific, Waltham, MA, United States) and 1% Triton X-100 (#NC9903183, Integra Chemical, Kent, WA, United States) in PBS (#10010023, Thermo Fisher Scientific, Waltham, MA, United States) for 1/2 h at room temperature, then immunostained overnight at room temperature with rabbit anti-myosin 7A (1:200, #25-6790, Proteus Biosciences, Ramona, CA, United States) diluted in 1% NHS (#16050130, Thermo Fisher Scientific, Waltham, MA, United States) with 0.4% Triton X-100 to label hair cells. After washing in PBS three times, cochlear pieces were incubated in Alexa Fluor 647-conjugated chicken anti-rabbit antibody at 1:200 (#A21443, Thermo Fisher Scientific, Waltham, MA, United States) and phalloidin at 1:200 (#A22283, Thermo Fisher Scientific, Waltham, MA, United States). A cochlear frequency map was created by imaging specimens at low magnification (10X objective) using a fluorescence microscope (E800, Nikon, Tokyo, Japan), and then applying a custom ImageJ plug-in developed at Massachusetts Eye and Ear^1^. Cochlear whole mounts were subsequently imaged with a confocal microscope (SP5, Leica, Wetzlar, Germany) and a glycerol-immersion 63X objective (1.3 N.A.) at log-spaced cochlear frequency regions from 4 to 64 kHz.

### RNA Isolation, cDNA Synthesis and Real-Time Quantitative Reverse Transcription Polymerase Chain Reaction (qRT-PCR)

Six hours after noise exposure, cochleae were extracted after decapitating deeply anesthetized mice and cochlear soft tissue was microdissected away from the bony otic capsule with fine forceps in RNAlater (Ambion, Austin, TX, United States). One specimen consisted of both cochleae from one animal. Total RNA was extracted using RNeasy Micro Kit (Qiagen GmbH, Hilden, Germany) and reverse transcribed with TaqMan Reverse Transcription Reagents kit (Applied Biosystems, Foster City, CA, United States) according to the manufacturers’ protocols. Real-time quantitative RT-PCR was performed using an Applied Biosystems 7700 Sequence Detection System and TaqMan Primers for *Coch* (Mm00483360_m1), *Adamts4* (Mm00556068_m1), *Adamts5* (Mm00478620_m1), *Il6* (Mm00446190_m1), *Tnf* (Mm00443258_m1), *Il1b* (Mm008409 04_m1), *Pycard* (Mm00445747_g1), *Cxcl1* (Mm04207460_m1), *Casp1* (Mm00438023_m1), *Nlrp3* (Mm00840904_m1), and reference gene 18S ribosomal RNA (Hs99999901_s1). Expression changes were analyzed by relative quantification and plotted as fold-change using 2^–ΔΔ*CT*^ method (Livak and Schmittgen, 2001; Stankovic and Corfas, 2003).

### Perilymph Collection

Perilymph was sampled 6 h after noise exposure using the approach adapted by our laboratory in a terminal procedure via the posterior semicircular canal (PSCC) (Landegger et al., 2019). In brief, mice anesthetized with ketamine (100 mg/kg i.p.) and xylazine (10 mg/kg i.p.) were given a booster as needed at half of the initial dose. A retroauricular stereotaxic approach permitted the exposure of the PSCC, which was opened after appropriate preparation. Sampling was achieved with the help of calibrated disposable micropipettes (VWR, Radnor, PA, United States) and three specimens per ear were collected (0.5 μL for vestibular perilymph = vPLF; 0.5 μL for cochlear perilymph = cPLF; up to 5 μL of cerebrospinal fluid = CSF). We observed minimal amounts of cochlin (data not shown) in CSF, suggesting some perilymph residue in the CSF sample. Only clear specimens were quantified and stored in an Eppendorf tube (Eppendorf, Hamburg, Germany) in 10 μL of double-filtered PBS (Millipore Sigma, Burlington, MA, United States). Tubes were sealed and placed into a −80°C freezer until further use.

### Perilymph Analysis

As described earlier (Landegger et al., 2019), the V-PLEX Pro-inflammatory Panel 1 Mouse Kit (#K15048D, Meso Scale Discovery = MSD, Rockville, MD, United States), an immunoassay based on electrochemiluminescence, was used to analyze the murine perilymph. Manufacturer’s instructions were followed, and the levels of the following cytokines were measured on a matching MSD SI2400 instrument by fitting electrochemiluminescence signals to the calibration curve: IFN-γ, IL-1β, IL-2, IL-4, IL-5, IL-6, CXCL1, IL-10, IL-12p70, and TNF-α.

### Western Blot

Western blot of cochlear perilymph was performed on perilymph samples collected through the PSCC as described above. Proteins were separated by SDS gel electrophoresis using reducing 4–12% polyacrylamide gradient gels and transferred to Immobilon-P (Millipore, Bedford, MA, United States) membranes using a wet transfer apparatus (Invitrogen, Carlsbad, CA, United States). Membranes were incubated with primary antibody monoclonal anti-cochlin (EMD Millipore, clone 9A10D2, Cat# MABF267) at 1:1,000 dilution. Blots were subjected to radiography after secondary antibody and PBST (Tween) washes.

The relative protein quantification of LCCL fragments was performed with ImageJ software (National Institutes of Health, MD, United States) (Davarinejad, 2018). Briefly, gel and western blotting PVDF membrane were imaged and analyzed using ImageJ after exporting images at 600 dpi in TIFF file format. Raw images were converted into a JPEG file format using Adobe Photoshop CS2 software (Adobe Systems Inc., San Jose, CA, United States) and changed to the grayscale picture mode. Using ImageJ tools, a single region of interest was created covering the minimum area containing the whole content of the largest band. This process was applied for each row of protein bands for each lane. The same frame was used for all of the protein bands across other lanes. Using ImageJ tools, density of the protein bands, including the background of empty fields, was converted into the pixel density, and exported into Excel (Microsoft Office, Microsoft Corporation, Redmond, WA, United States). Inversion of pixel density was performed using subtraction of the recorded values from 255. The net value for all protein bands was determined by deducting the inverted background from the inverted band values. Relative quantification values were expressed as a ratio of the net band values of LCCL fragments in each sample to the sum of the net bands of all proteins in the sample (the total protein value).

### Statistical Analysis

**FIGURE 1:**
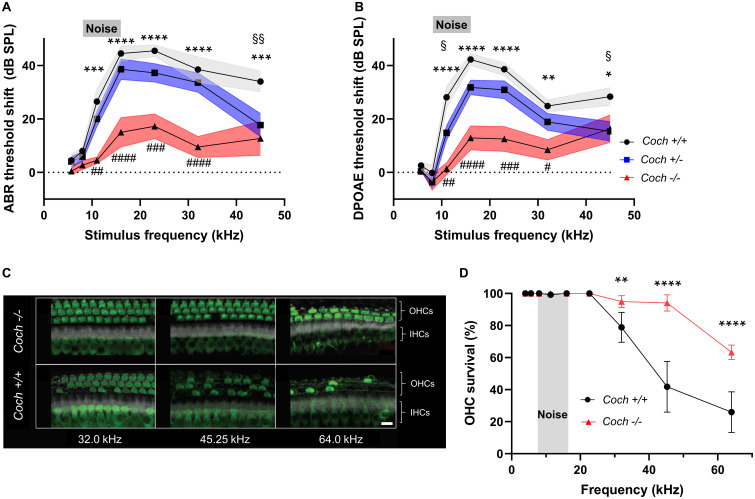
Absence of cochlin reduces the level of cochlear dysfunction and sensory cell damage after acoustic injury. Six-week-old mice of each genotype were exposed to 8–16 kHz noise for 2 h at 103 dB SPL. **(A)** ABR and **(B)** DPOAE threshold shifts 2 weeks after noise trauma demonstrate profound mid-to-high frequency hearing loss in wild-type *Coch*^+/+^ mice, and statistically significant lower threshold shifts in *Coch*^–/^*^–^* mice. A trend toward lower threshold shifts is observed in heterozygous mice, which reaches statistical significance at 11.33 kHz (DPOAE) and 45.25 kHz (ABR and DPOAE). The gray rectangle indicates frequency of noise band. Data are shown as group means ± standard error of the mean; *N* = 10 *Coch*^+/+^, *N* = 11 *Coch*^+/–^, and *N* = 11 *Coch*^–/^*^–^* animals. *P < 0.05, **P < 0.01, ***P < 0.001, or ****P < 0.0001; asterisks: *Coch*^+/+^ vs. *Coch*^–/^*^–^*, #: *Coch*^+/–^. *Coch*^–/^*^–^*, §: *Coch*^+/–^ vs. *Coch*^+/+^. **(C)** Representative cochlear whole mounts from *Coch*^+/+^ and *Coch*^–/^*^–^* mice 2 weeks after acoustic trauma. IHC, inner hair cells. OHC, outer hair cells. Green = myosin 7A, white = phalloidin. Scale bar: 10 μm. **(D)** Cochleogram showing fewer missing outer hair cells in *Coch*^–/^*^–^* mice 2 weeks after acoustic trauma. Data are shown as group means ± standard error of the mean; *N* = 4 *Coch*^+/+^ and *N* = 4 *Coch*^–/^*^–^* mice. **P < 0.01 or ****P < 0.0001.

**FIGURE 2:**
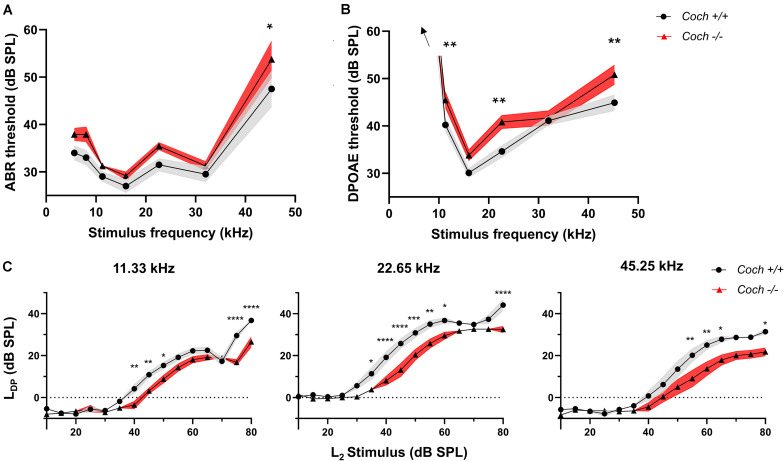
Cochlin knock-out mice have small but significant elevations in audiometric thresholds compared to wild-type mice. **(A)** ABR and **(B)** DPOAE thresholds are higher in *Coch*^–/^*^–^* compared to *Coch*^+/+^ mice. The ABR threshold difference reaches statistical significance at 45.25 kHz. DPOAE threshold difference reaches significance at 11.33, 22.65, and 45.25 kHz. Thresholds at 5.66 and 8 kHz >60 dB SPL. **(C)** Input/output functions of DPOAEs at 11.33 kHz, 22.65 kHz, and 45.25 kHz show a right shift and largely unaltered growth function in *Coch^–/–^* animals compared to *Coch*^+/+^. Data are shown as group means ± standard error of the mean; *N* = 10 *Coch*^+/+^ and *N* = 12 *Coch*^–/^*^–^* animals. *P < 0.05, **P < 0.01, ***P < 0.001, ****P < 0.0001. L_*DP*_ = DPOAE level, L_2_ = primary tone level.

**FIGURE 3:**
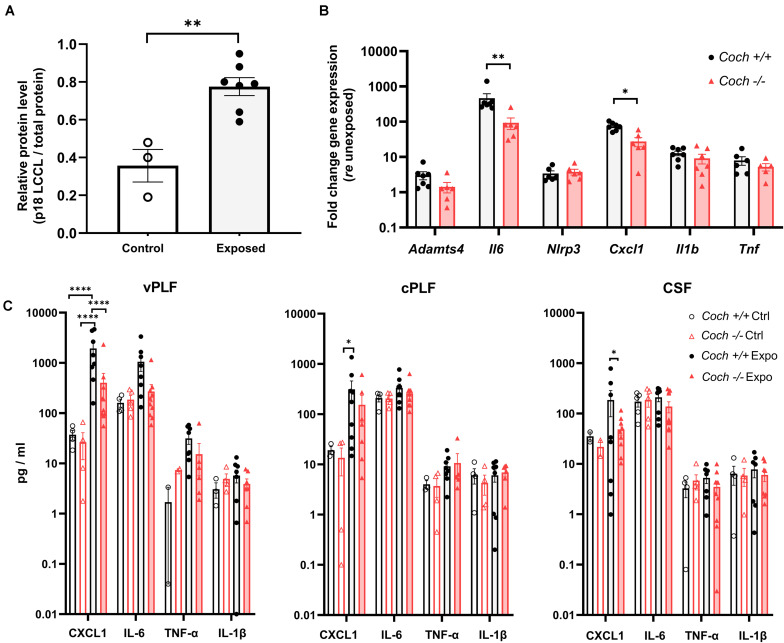
Cochlin-deficient mice demonstrate reduced pro-inflammatory response to acoustic trauma in cochlear soft tissue and perilymph. Six-week-old *Coch^–/–^* and *Coch*^+^*^/^*^+^ mice were exposed to 8–16 kHz noise for 2 h at 103 dB SPL. Unexposed mice served as controls. **(A)** Six hours post exposure, cochlear perilymph of wild-type animals demonstrated significantly increased levels of the cleaved LCCL domain (18 kDa fragment) compared to unexposed animals. Data are shown as group means of relative protein level of 18 kDa LCCL fragment (p18) ± standard error of the mean. *^∗∗^P* < 0.01 (unpaired *t*-test). Cochlear perilymph was collected through the posterior semicircular canal. Expression of LCCL fragment was determined by Western blotting using an anti-cochlin monoclonal antibody. Protein level of LCCL fragment was calculated as a ratio of LCCL p18 protein bands relative to the total protein bands. *N* = 3 ears from unexposed mice, 7 ears from exposed mice. **(B)** Cochleae collected 6 h post exposure had statistically significant elevation of *Il6* and *Cxcl1* gene expression in *Coch*^+^*^/^*^+^ compared to *Coch^–/–^* mice, and demonstrated a similar trend that did not meet our criterion for significance for *Adamts4, Il1b*, and *Tnf*. *N* = 7 animals per group. Data are shown as group means ± standard error of the mean. *^∗^P* < 0.05, *^∗∗^P* < 0.01. **(C)** Six hours post exposure, perilymph demonstrated significantly lower CXCL1 levels in *Coch^–/–^* compared to *Coch*^+^*^/^*^+^ mice and trends toward lower IL-6, TNF-α, and IL-1β levels, that did not meet our criterion of statistical significance. Data are shown as group means ± standard error of the mean. *^∗^P* < 0.05, ^****^P < 0.0001. Vestibular perilymph (vPLF), cochlear perilymph (cPLF), and cerebrospinal fluid (CSF) were collected through the posterior semicircular canal. *N* = 4 *Coch*^+^*^/^*^+^ unexposed ears, 5 *Coch*^+^*^/^*^+^ exposed ears, 9 *Coch^–/–^* unexposed ears, and 10 *Coch^–/–^* exposed ears.

Statistical analysis was performed using GraphPad Prism 9.0.0 (GraphPad Software, Inc., San Diego, CA, United States). Statistical significance in ABR and DPOAE data (thresholds, I/O-functions) (Figures 1A,B, 2) as well as cytokine expression in perilymph (Figure 3C) was determined using ordinary two-way ANOVA and subsequent Tukey’s multiple comparisons test. For outer hair cell-loss, ordinary two-way ANOVA with subsequent Šidak test for multiple comparisons was employed (Figure 1D). qPCR data were analyzed by unpaired *t*-test with Holm-Šidak method for multiple comparisons (Figure 3B and Supplementary Figures 2A,B). Western blot of perilymph was analyzed by unpaired *t*-test (Figure 3A). A probability value of *P* < 0.05 was considered statistically significant. All data are presented as means ± standard errors of the mean (SEMs).

## Results

### Protection From Permanent Noise Trauma in *Coch^–/–^* Mice

Sound entering the inner ear causes vibration of the basilar membrane, which is amplified by OHCs, leading to stimulation of inner hair cells, neurotransmitter release, and transmission of electrical signals via the auditory nerve to the brain. DPOAEs are produced by OHCs not only because cochleae amplify vibrations, but also because they exhibit non-linearity (Hudspeth, 2014). The ABR captivates the neural response to sound, initiated at the inner hair cell-spiral ganglion neuron synapse. While a previous study showed mild physiological changes (slightly elevated ABR threshold only in the highest frequency tested, in older mice) but no morphological cochlear deficits in *Coch^–/–^* mice (Jones et al., 2011), here we investigated how *Coch*^–/^*^–^* mice would respond to an external challenge, in this case high noise exposure. For these measurements, we exposed 6-week-old littermates of three genotypes – wild-type *Coch*^+^*^/^*^+^ (*N* = 10) mice, heterozygous *Coch*^+/–^ (*N* = 10) mice and homozygous *Coch^–/–^* (*N* = 11) mice – to 8–16 kHz octave-band noise at 103 dB SPL for 2 h. We have previously shown that these noise parameters cause permanent hearing loss accompanied by outer hair cell loss in the cochlear basal turn and substantial intracochlear inflammation (Landegger et al., 2019). Hearing thresholds were measured by ABR (Figure 1A) and DPOAE (Figure 1B) 2 weeks after noise exposure and demonstrated threshold elevations in all genotypes. To our surprise, compared to the wild-type genotype, *Coch^–/–^* mice had significantly lower ABR threshold shifts (Figure 1A) at 11.33 kHz (mean difference = 21.95 dB SPL, *P* = 0.0001), 16.00 kHz (mean difference = 29.50 dB SPL, *P* < 0.0001), 22.65 kHz (mean difference = 28.23 dB SPL, *P* < 0.0001), 32.00 kHz (mean difference = 28.9 dB SPL, *P* < 0.0001), and 45.25 kHz (mean difference = 21.27 dB SPL, *P* = 0.0001), showing a protective effect to noise as compared to wild-type mice. *Coch*^+/–^ mice demonstrated a trend toward lower threshold shifts than wild-type mice, which reached statistical significance at 45.25 kHz (mean difference = 16.27 dB SPL, *P* = 0.0049). Compared to wild-type mice, DPOAE threshold shifts (Figure 1B) in *Coch^–/–^* mice were significantly lower at 11.33 kHz (mean difference = 26.74 dB SPL, *P* < 0.0001), 16.00 kHz (mean difference = 29.39 dB SPL, *P* < 0.0001), 22.65 kHz (mean difference = 26.05 dB SPL, *P* < 0.0001), 32.00 kHz (mean difference = 16.45 dB SPL, *P* = 0.0010), and 45.25 kHz (mean difference = 12.04 dB SPL, *P* = 0.0227). *Coch*^+/–^ mice showed a trend toward lower DPOAE threshold shifts than wild-type mice, which reached significance at 11.33 kHZ (mean difference = 13.28 dB SPL, *P* = 0.0103) and 45.25 kHz (mean difference = 13.04 dB SPL, *P* = 0.0121). Sex-specific analysis did not show any major differences between female and male animals (Supplementary Figure 1).

Next, we assessed morphological changes in cochlear whole mounts from noise-exposed wild-type and *Coch*^–/^*^–^* mice to identify structural evidence for the observed functional outcome. Hair cells were identified using phalloidin stain and antibodies to Myo7A as hair cell marker. The number of surviving outer hair cells (OHCs) in *Coch*^–/^*^–^* mice was higher than in wild-type animals across the frequencies of maximal noise susceptibility, as illustrated in representative images in Figure 1C. Acoustic injury typically causes maximal damage at frequencies higher than the stimulus frequency (Cody and Johnstone, 1981). This upward spread of cochlear damage arises because of level-dependent non-linearities in cochlear mechanics (Robles and Ruggero, 2001). The qualitative observation illustrated in Figure 1C was supported by quantitative measurements (Figure 1D) demonstrating that *Coch*^–/^*^–^* animals had significantly higher OHC counts at 32.00 kHz (*P* = 0.0013), 45.25 kHz (*P* < 0.0001), and 64.00 kHz (*P* < 0.0001). Taken together, cochlin-lacking mice exhibited a greater survival of OHCs than their wild-type littermates after noise exposure, which led to reduced ABR and DPOAE thresholds.

### *Coch^–/–^* Mice Exhibit Signs of Mild Conductive Hearing Loss

To determine whether cochlin deficiency confers protection from noise-induced hearing loss via a conductive or sensorineural mechanism, we measured audiometric thresholds in littermates of *Coch^–/–^* and *Coch*^+^*^/^*^+^ mice. To maximize robustness and reproducibility of the data, all measurements were performed by a researcher blinded to the genotype. Six-week-old unexposed *Coch^–/–^* mice (*N* = 12) demonstrated two to six dB higher ABR (Figure 2A) and DPOAE (Figure 2B) thresholds compared to *Coch*^+^*^/^*^+^ mice (*N* = 10). This difference was statistically significant in ABR thresholds at 45.25 kHz (*P* = 0.0252) and DPOAE thresholds at 11.33 kHz (*P* = 0.0187), 22.65 kHz (*P* = 0.0043), and 45.25 kHz (*P* = 0.0071). *Coch*^+^*^/^*^+^ and *Coch*^+/–^ animals (*N* = 13) were statistically indistinguishable (data not shown).

To assess a possible mechanical contribution to the elevated thresholds in *Coch*^–/^*^–^* mice, we further evaluated DPOAE input/output functions. Cochlear dysfunction is known to lead to a change in slope of the growth function, whereas middle ear pathologies cause a right shift of the curve with unaltered slope of the growth function (Gehr et al., 2004). Input/output functions of DPOAEs at 11.33 kHz, 22.65 kHz, and 45.25 kHz show a statistically significant right shift and largely unaltered slope of the growth function in *Coch^–/–^* animals compared to *Coch*^+^*^/^*^+^ animals (Figure 2C), with the exception of a slight reduction in slope at 45.25 kHz. Taken together, these data are consistent with a small conductive hearing loss in *Coch^–/–^* mice compared to *Coch*^+^*^/^*^+^ and a possible additional sensorineural component at the highest tested frequency.

### Noise Trauma Causes Weaker Pro-inflammatory Response in *Coch^–/–^* Mice

To further study the mechanism behind the protection from noise exposure in cochlin-deficient animals, we collected perilymph from the posterior semicircular canal 6 h after noise exposure, because we previously reported a robust immune reaction at that time point (Landegger et al., 2019). Six hours after exposure of *Coch*^+^*^/^*^+^ mice to 103 dB noise, we detected an increase in the level of cleaved LCCL domain p18 (18 kDa fragment) compared to unexposed *Coch*^+^*^/^*^+^ mice (*P* = 0.0018), as assessed by western blot of cochlear perilymph (Figure 3A and Supplementary Figure 3). We also found the expression of the gene *Adamts4* encoding the metalloprotease enzyme, aggrecanase1 responsible for cleaving the cochlin LCCL, and initiating immune response (Py et al., 2013), to be elevated after noise exposure. *Adamts4* expression as measured by quantitative real-time PCR, was upregulated in cochlear soft tissue 6 h after 103 dB noise in *Coch*^+^*^/^*^+^ and *Coch^–/–^* mice; *Coch^–/–^* mice showed a trend toward lower *Adamts4* expression than *Coch*^+^*^/^*^+^ mice after noise exposure (Figure 3B). The expression of genes encoding the pro-inflammatory cytokines IL-6 (*P* = 0.005881) and CXCL1 (*P* = 0.032899) were significantly elevated in *Coch*^+^*^/^*^+^ mice compared to *Coch^–/–^* mice. A similar trend was demonstrated for *Il1b* and *Tnf* genes (Figure 3B) although it did not meet our criterion for significance. Inflammasome-associated genes were significantly upregulated in exposed wild-type cochleae: *Il1b (P* = *0.002001*), and *Nlrp3* (*P* = *0.008397*) (Supplementary Figure 2A). A trend toward reduced activation of inflammasome-associated genes in *Coch^–/–^* compared to *Coch*^+^*^/^*^+^ cochleae was measured 6 h after noise exposure (Supplementary Figure 2B). In vestibular perilymph, we observed significantly lower CXCL1 levels in *Coch^–/–^* compared to *Coch*^+^*^/^*^+^ mice (*P* < 0.0001) and trends for lower levels of IL-6, IL-1β, and TNF-α that did not meet our criterion for statistical significance (Figure 3C).

## Discussion

Using a combination of physiological tests that assess cochlear function, here we demonstrate for the first time that *Coch^–/–^* mice have a small conductive hearing loss compared to *Coch*^+^*^/^*^+^ littermates, and possibly additional sensorineural component at the highest tested frequency. Together with the previously reported expression of cochlin in the murine and human middle ear, precisely on the articular surfaces of the incudomalleal and incudostapedial joints as well as pars tensa of the tympanic membrane (Robertson et al., 2014), we highlight cochlin’s importance in sound conduction across the middle ear. In previous studies, a trend for increased ABR thresholds was present in young *Coch^–/–^* mice compared to *Coch*^+^*^/^*^+^ mice, although this trend did not reach the authors’ criterion for significance (Choe et al., 2019). The small number of mice tested in that study, four per group, may have prevented detection of a statistically significant change. In contrast, by more than doubling the number of mice in our cohorts, we were able to detect a statistically significant elevation in ABR and DPOAE thresholds due to cochlin deficiency.

Precisely quantifying the degree of conductive hearing loss due to cochlin deficiency is important in this era of emerging inner ear gene therapy, as correction of *COCH* mutations via localized delivery to the inner ear will leave the middle ear unaddressed. Long-term conductive hearing loss is known to lead to speech intelligibility deficits in patients and loss of synapses in an animal model of conductive hearing loss (Liberman et al., 2015; Okada et al., 2020). Future murine studies should therefore quantify air- and bone-conduction evoked ABRs (Qin et al., 2010; Chhan et al., 2017) to further delineate conductive vs. sensorineural contribution to HL in *Coch^–/–^* mice. In addition, future clinical studies should evaluate for potential presence of conductive hearing loss in patients with *COCH* pathogenic variants.

In the meantime, our finding that noise exposure in wild-type mice causes an increase of the pro-inflammatory LCCL component of cochlin in perilymph compared to unexposed controls is significant because it is consistent with noise-causing sterile inflammation. It is known that inflammatory stimuli such as lipopolysaccharide (LPS) and TNF-α, and pathogens including *Pseudomonas aeruginosa* and *Staphylococcus aureus* lead to post-translational cleavage of full-length cochlin (p60, 60 kD) between the LCCL and vWFA1 domains by aggrecanase1. This, in turn, releases the cochlin-LCCL self-folding modular protein isoform (p8-p16, 8 kD–16 kD, including glycosylated forms) into the bloodstream or perilymph. This process results in local downstream effects on inflammatory cytokine production, macrophage activation, and immune cell recruitment in the infected organ (Moussion and Sixt, 2013; Py et al., 2013; Nyström et al., 2018; Choe et al., 2019; Komatsu and Li, 2019; Rhyu et al., 2020).

The first and typically irreversible cellular changes after noise exposure are degeneration of spiral ligament fibrocytes (Kujawa and Liberman, 2006) and cochlear afferent neurites (Kujawa and Liberman, 2009). Whereas there is abundant cochlin expression in the spiral ligament, there is no immunohistochemically detectable cochlin in spiral ganglion cell bodies and neurites (Robertson et al., 2006). However, cochlin is immunohistochemically detectable in cells lining Rosenthal’s canal and the osseus spiral lamina channels that surround the neural compartments of the cochlea (Robertson et al., 2006). Cochlin’s location surrounding neural tissue warrants future studies to determine whether cochlin plays a role in cochlear neuropathy and synaptopathy. The importance of cochlin for spiral ligament function is demonstrated by histopathological analysis of human DFNA9 temporal bones that show cell loss and replacement with eosinophilic acellular material in the spiral ligament, as well as the spiral limbus, and stroma of the cristae and maculae (Robertson et al., 1998, 2006; Merchant et al., 2000). We show for the first time that cochlin’s LCCL component is cleaved after intense noise exposure through aggrecanase1 and released into the perilymph. It is likely that this LCCL component is cleaved from the spiral ligament extracellular matrix given the abundance of cochlin in the spiral ligament and the spiral ligament’s central role in inflammatory responses (Adams, 2002). Precise mechanisms of LCCL entry into the perilymph and how this affects patients suffering from DFNA9, noise, and age-related hearing loss remains to be determined in the future.

Due to the apparent and surprising conductive hearing loss in *Coch* knock-out mice, the observed protection from noise in these *Coch^–/–^* mice cannot be entirely attributed to the lack of LCCL cleaving in the inner ear and its downstream reduction of inflammatory cytokines. It is, however, possible that the mild conductive loss attenuates the amount of noise carried into the cochlea, thus preventing some of the noise-induced damage. Strategically designed additional experiments are needed to dissect out contributions from cochlin deficiency in the inner ear and cochlin deficiency in the middle ear as the latter causes conductive hearing loss and effectively reduces noise input to the inner ear. These future experiments could involve mouse models lacking cochlin in the inner ear only or middle ear only, as well as pharmacologic (Durham et al., 2017) or genetic inhibition of aggrecanases.

## Data Availability Statement

The original contributions presented in the study are included in the article/Supplementary Material, further inquiries can be directed to the corresponding author.

## Ethics Statement

The animal study was reviewed and approved by the Institutional Animal Care and Use Committee of Massachusetts Eye and Ear.

## Author Contributions

KS, LL, and RS conceived and planned the experiments. LL, RS, NR, and SV performed the experiments and analyzed the data. KS, RS, and NR wrote the manuscript with inputs from all authors. KS supervised all aspects of research. All authors contributed to the article and approved the submitted version.

## Conflict of Interest

The authors declare that the research was conducted in the absence of any commercial or financial relationships that could be construed as a potential conflict of interest.

## Footnotes

1 https://www.masseyeandear.org/research/otolaryngology/eaton-peabody-laboratories/histology-core

## Supplementary Material

Supplementary Figure 1 Protection from noise-induced hearing loss in *Coch^–/–^* compared to *Coch^+/+^* mice is similar in both sexes. Replotting of results depicted in Figure 1. Six-week-old mice of each genotype were exposed to 8–16 kHz noise for 2 h at 103 dB SPL; ABR threshold shifts were measured 2 weeks later. The gray rectangle indicates frequency of noise band. There was no significant difference between the sexes of each genotype. *N* = 3 *Coch*^+/+^ males, *N* = 7 *Coch*^+/+^ females, *N* = 5 *Coch*^–/^*^–^* males, and *N* = 6 *Coch*^–/^*^–^* females.

Supplementary Figure 2 Acoustic trauma leads to upregulation of inflammasome-associated genes in WT cochleae and a trend toward reduced activation of inflammasome-associated genes in *Coch^–/–^* compared to *Coch^+/+^* cochleae. Six-week-old *Coch*^+/+^ and *Coch*^–/^*^–^* mice were exposed to 8–16 kHz noise for 2 h at 103 dB SPL. Unexposed mice served as controls. **(A)** Wild-type cochleae collected 6 h post exposure (gray bars) had statistically significant elevation in expression of *Nlrp3* and *Il1b* genes compared to unexposed (white bars) cochleae and demonstrated a similar trend for *Casp1*. Data are shown as group means ± standard error of the mean. *^∗∗^P* < 0.01. **(B)** Six hours post exposure, *Coch*^–/^*^–^* cochleae showed a trend toward reduced expression of inflammasome-associated genes; this trend did not meet our criterion for significance. *N* = 7 mice per group.

Supplementary Figure 3 Raw western blot data used for quantification presented in Figure 3A. Six-week-old *Coch*^+^*^/^*^+^ mice were exposed to 8–16 kHz noise for 2 h at 103 dB SPL. Unexposed mice served as controls. Six hours post exposure, perilymph was collected through the posterior semicircular canal. Left, representative Ponceau stained gel. Right, corresponding PVDF membrane stained with anti-cochlin monoclonal antibody. The anti-cochlin antibody identified strong protein bands of 18 kDa LCCL fragment in perilymph of noise-exposed animals (lanes 4–10) (*N* = 7 ears) and weaker bands of the same fragment in perilymph of control animals (lanes 1–3) (*N* = 3 ears). The arrows point to 18 kDa (p18), 16 kDa (p16), and 8 kDa (p8) LCCL-specific bands.
