# Supplementary figures and images for: Cochlin Deficiency Protects Against Noise-Induced Hearing Loss
Source: Front Mol Neurosci. 2021 May 24;14:670013. doi: 10.3389/fnmol.2021.670013 (PMC8180578; doi:10.3389/fnmol.2021.670013)

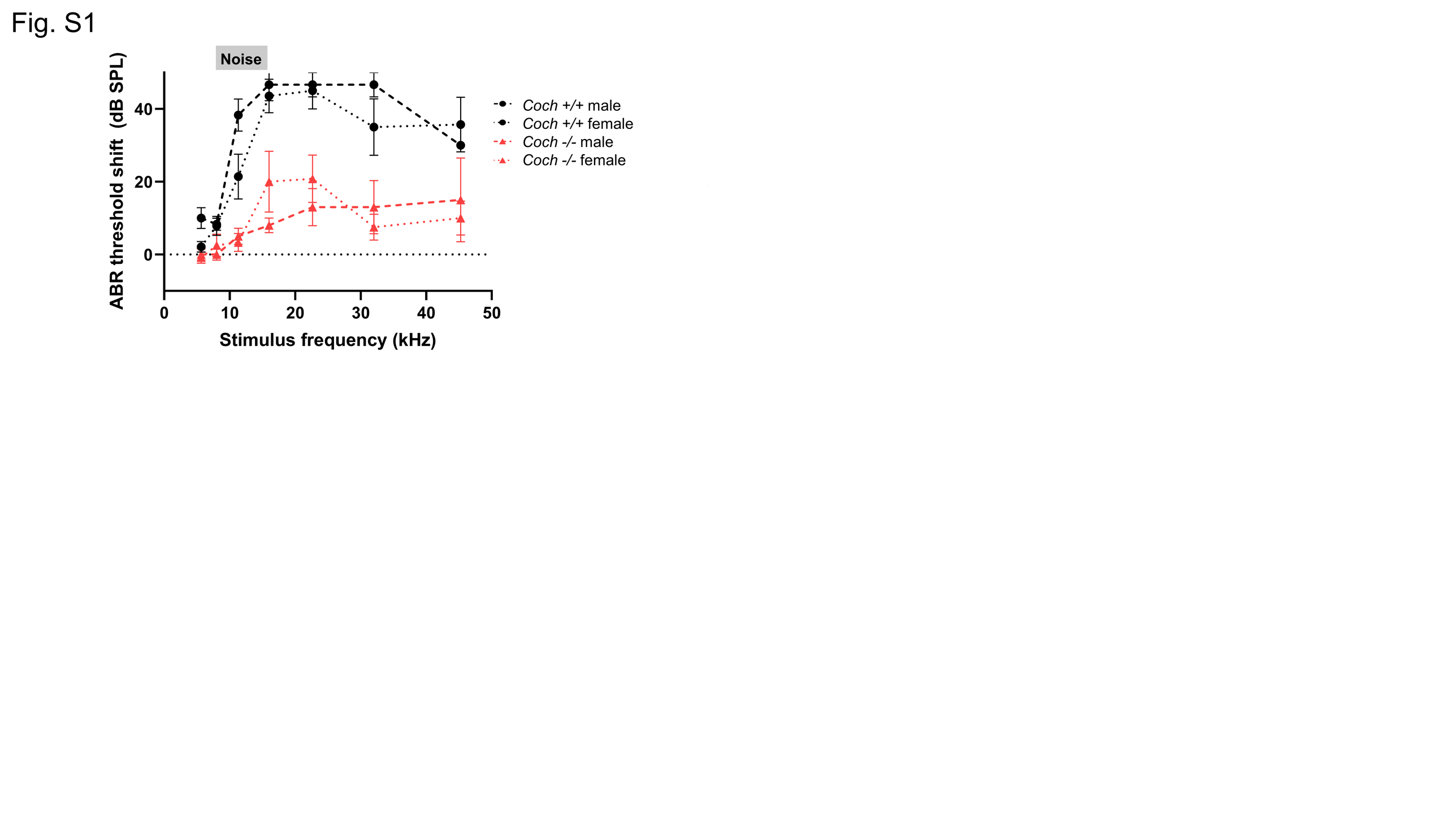

Supplement: Supplementary Figure 1 — Protection from noise-induced hearing loss in Coch–/– compared to Coch+/+ mice is similar in both sexes. Replotting of results depicted in Figure 1. Six-week-old mice of each genotype were exposed to 8–16 kHz noise for 2 h at 103 dB SPL; ABR threshold shifts were measured 2 weeks later. The gray rectangle indicates frequency of noise band. There was no significant difference between the sexes of each genotype. N = 3 Coch+/+ males, N = 7 Coch+/+ females, N = 5 Coch–/– males, and N = 6 Coch–/– females. [file Image_1.tif]

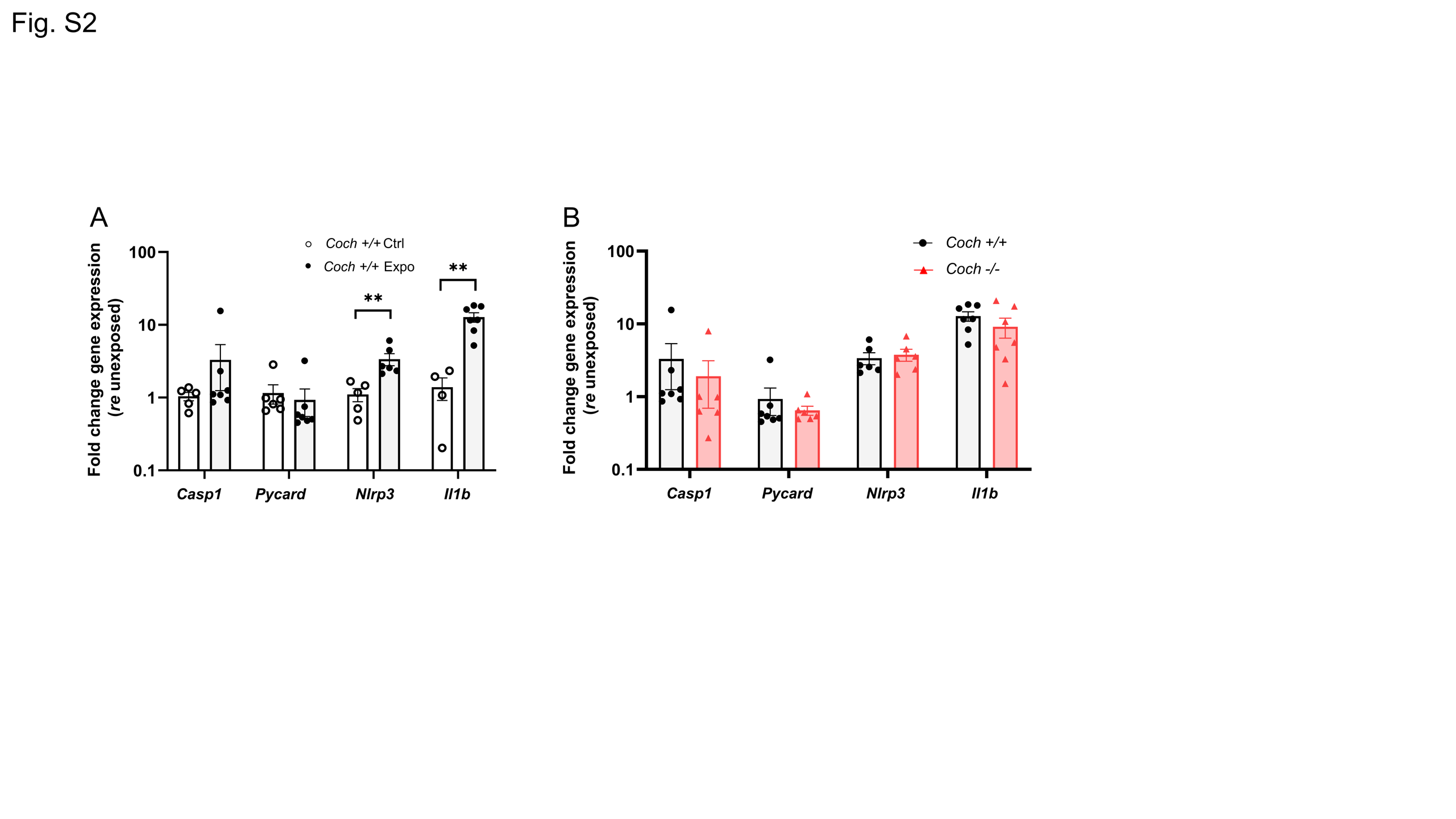

Supplement: Supplementary Figure 2 — Acoustic trauma leads to upregulation of inflammasome-associated genes in WT cochleae and a trend toward reduced activation of inflammasome-associated genes in Coch–/– compared to Coch+/+ cochleae. Six-week-old Coch+/+ and Coch–/– mice were exposed to 8–16 kHz noise for 2 h at 103 dB SPL. Unexposed mice served as controls. (A) Wild-type cochleae collected 6 h post exposure (gray bars) had statistically significant elevation in expression of Nlrp3 and Il1b genes compared to unexposed (white bars) cochleae and demonstrated a similar trend for Casp1. Data are shown as group means ± standard error of the mean. ∗∗P < 0.01. (B) Six hours post exposure, Coch–/– cochleae showed a trend toward reduced expression of inflammasome-associated genes; this trend did not meet our criterion for significance. N = 7 mice per group. [file Image_2.tif]

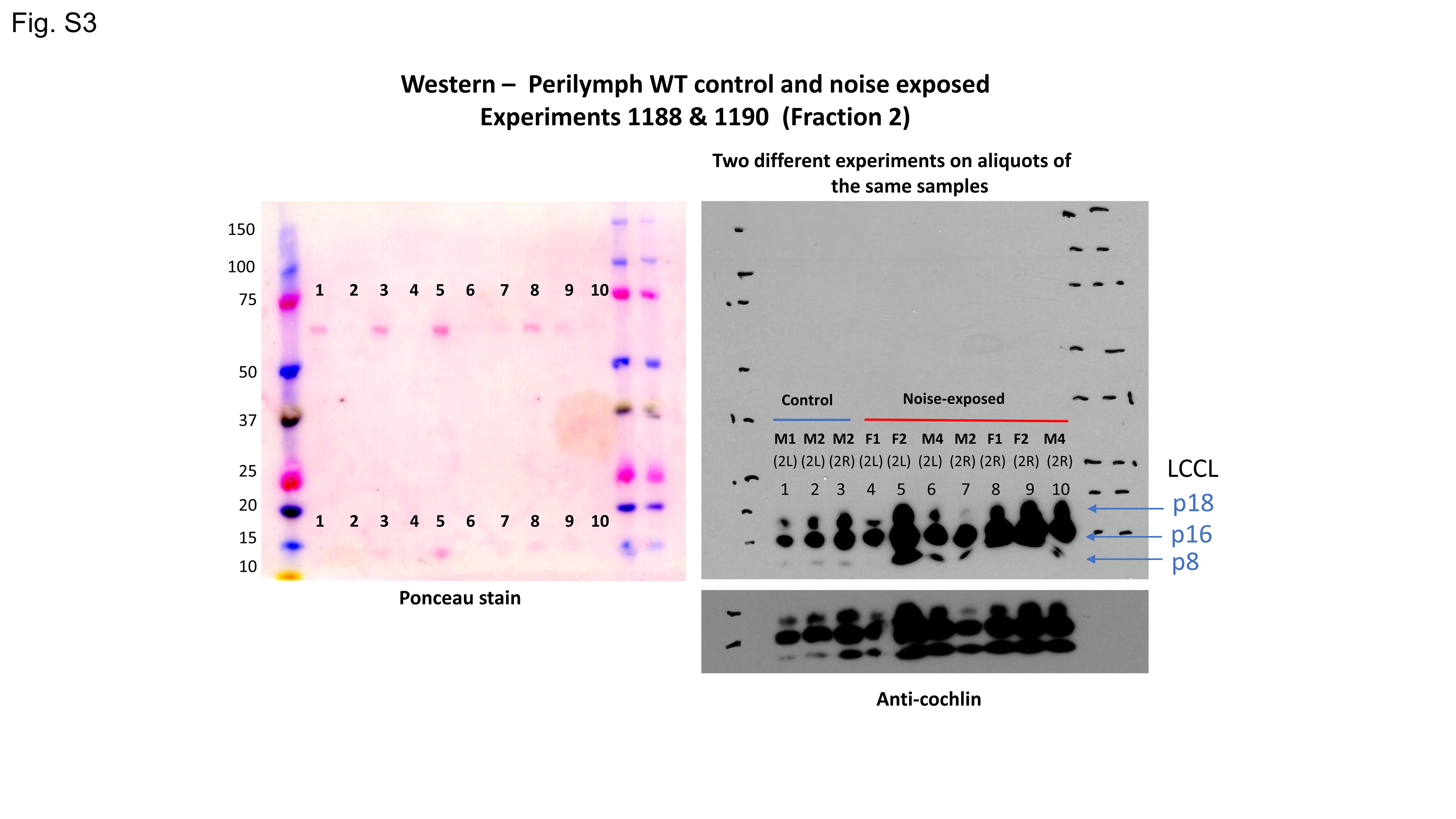

Supplement: Supplementary Figure 3 — Raw western blot data used for quantification presented in Figure 3A. Six-week-old Coch+/+ mice were exposed to 8–16 kHz noise for 2 h at 103 dB SPL. Unexposed mice served as controls. Six hours post exposure, perilymph was collected through the posterior semicircular canal. Left, representative Ponceau stained gel. Right, corresponding PVDF membrane stained with anti-cochlin monoclonal antibody. The anti-cochlin antibody identified strong protein bands of 18 kDa LCCL fragment in perilymph of noise-exposed animals (lanes 4–10) (N = 7 ears) and weaker bands of the same fragment in perilymph of control animals (lanes 1–3) (N = 3 ears). The arrows point to 18 kDa (p18), 16 kDa (p16), and 8 kDa (p8) LCCL-specific bands. [file Image_3.tif]
